# Oral Sepsis and Its Relation to Abdominal Disease

**Published:** 1908-02-08

**Authors:** John H. Dauber

**Affiliations:** Gynæcologist to the Hospital for Women, Soho.


### Hospital Clinics.

### ORAL SEPSIS AND ITS RELATION TO ABDOMINAL DISEASE.

By JOHN H. DAUBER, M.A., M.B., B.Ch., (Oxon), Gynæcologist to the Hospital for Women, Soho.

(A Clinical Lecture delivered at the Hospital on January 22.)

Not infrequently it happens that the obvious is overlooked. To stumble over some obstacle in our path while scanning the horizon is no uncommon mistake, and so it sometimes falls out that while looking far afield for the causation of some obscure abdominal symptom, we fail to take cognisance of important factors lying literally under our very noses.

Since my connection with the out-patient department of this hospital my attention has been called again and again, by constantly recurring coincidences, to the great influence exercised over the entire organism by the condition of the buccal cavity. Much has been written and said on the subject of Oral Sepsis recently, and the address of Mr. William Hern to the students of the Middlesex Hospital a few years ago is, amongst several monographs which have more lately appeared on the subject, one of conspicuous value, and worthy of being read and re-read by everyone engaged in the care and treatment of the sick. As, however, life is too full for everyone to read all that is written, a great deal of careful observation must needs be wasted, and so, in spite of constant reminders, the importance of examining the condition of the teeth, gums and fauces in the case of every patient, is not even yet sufficiently appreciated. It is only when the mouth is scrutinised in every case, without exception, as a matter of routine, that it begins to dawn upon the student or practitioner that good health is a matter of sheer impossibility in many of the cases he sees, owing to the neglected state in which the mouth is kept.

A certain well-known surgeon once caused some amusement by stating that he considered it to be the duty of the public authorities to impregnate the water of the drinking fountains in London with iodide of potassium, as he considered so few of the population were free from the taint of syphilis. Of the prevalence of the syphilitic poison there may be some doubt, but there can be no two opinions about the universality of dental caries amongst the people. If anyone should systematically look into the mouths of all the out-patients attending the London hospitals, he would be appalled by the state of affairs revealed, and would certainly form the opinion that dental hospitals should be forthwith established at every street corner to deal with the vast amount of caries that is prevalent. The present dental institutions are quite inadequate to deal with the mass of dental disease in London; dentists themselves admit it, and in my opinion there is no more urgent want at the present time in this city than institutions in superabundance where poor people should be able to get their teeth stopped, or extracted, or replaced with artificial ones, at a cost within their means. The duty of attending to and caring for the teeth should be a part of the routine instruction in all elementary schools. That it is not so we all know too well. The following is a case in point:—

A patient told me only a few days ago, when I noticed that several molars were missing in spite of the fact that she evidently paid much attention to her teeth and personal appearance, that it was only at the age of fifteen that she began to clean her teeth, and that she had lost all the missing teeth, without exception, prior to that date. It is only reasonable to believe in this case that if, as a child, she had been taught not to allow food to remain in the interstices between the teeth, but to brush them and rinse out the mouth as she has done since, that she would now have a complete set of teeth. The State, having usurped the parents' duties, should perform them. What can be done in this direction is evidenced by the results obtained under the Factory Act in the reduction of cases of "Phossy Jaw," which are now hardly seen.

There are about fifteen new cases daily at this hospital, and on my days I have noticed that it is most rare to find any patient without one or more septic teeth. Strictly as one may attempt to limit cases coming here to those of purely gynæcological interest, yet patients are unable to diagnose their own complaints. A certain percentage attend who either in conjunction with, or apart from pelvic mischief, are suffering from conditions attributable to deficient nutrition, general septic absorption, toxæmia, or some form of inflammation or colic of one or other portion of the alimentary canal. I will trouble you with a brief recital of a few cases to illustrate my meaning.

1. At the present moment there is in the Sydney Ward, No. 5, a patient, a married woman, specially sent up to the hospital with a doctor's letter of recommendation. In the absence of my senior colleague I examined her, and found the pelvis normal, with the sole exception that the uterus was slightly tilted backwards but quite movable. She complained of constant abdominal pain, from which she had suffered for many years past, together with loss of flesh. I examined her mouth, and made a note on her paper, "All teeth absent or septic." There were no teeth in the upper jaw at all, but many carious stumps. In the lower jaw a few teeth stood out here and there above the alveolus at irregular intervals, like posts; the rest of the lower jaw contained numerous septic roots. I concluded, and my opinion was confirmed by my colleague on his return, that the mal-nutrition and abdominal pain were due to the foul state of the portal to the nutrient canal, rather than to the trivial backward declination of the uterus, and this patient will be passed on to a dental hospital.

2. A short time ago we had a patient in Cadogan

Ward who had been an in-patient in the wards on two, if not three, previous occasions. We could find nothing really amiss in the pelvis, but as often as we sent her out her doctor advised her readmission, asserting, and truly, that his patient suffered as much from abdominal pain as ever before. We were at a loss to know what to do; the pain seemed a real thing, and exploratory laparotomy began to loom like a cloud on the horizon as a possible expedient for diagnosis and relief; but at the last examination of this patient it occurred to me to look inside her mouth, and there the same state of affairs as in the previous patient revealed itself—a collection of septic teeth, ragged stumps, and vacant spaces. The patient had absolutely nothing to masticate with; her food must have been swallowed in an insufficiently sub-divided state for the alimentary canal to deal with; moreover, with the food, undoubtedly, septic material must have been swallowed. To make a long story short, this patient made a good recovery after her mouth was put in order and artificial teeth supplied, when no more was heard of her abdominal pains.

3. A few months ago there was a patient in No. 1 bed, Shaftesbury Ward, who came in for removal of an ovarian cystoma. I had quite forgotten her, but she beckoned me to her side and reminded me that three years previously she had been in my outpatient department, and I had advised her to have her teeth attended to, and for this advice she wished to thank me. Unlike most patients, who dislike unpalatable advice, she had consulted a dentist to good purpose, having carious teeth extracted and an artificial set fitted to each jaw. Before this, she said, for 20 years, as girl and woman, she had suffered from chronic indigestion and pains in the stomach. All that time she had been "doctoring" and had taken bottles and bottles of physic, but of all the doctors she had seen she could not remember one who had looked at her teeth until she came to this hospital, nor had she ever consulted a dentist until then; since her teeth had been put right she had been a new woman.

4. A lady of my acquaintance last year was operated upon for appendicitis. The operation had to be undertaken at very short notice, the symptoms being urgent. The appendix was found to be distended with pus, but fortunately it had not ruptured. The operation was a complete success, the patient made an uninterrupted recovery, and at the end of three weeks was well enough to go for a drive. The day after this she became grievously ill, the temperature rose to 102° and 103°, several consultations were held, and one physician hazarded the conjecture that, as in previous years the patient had suffered from pulmonary phthisis, there might be a lighting-up or fresh dissemination of the old tubercular trouble. The surgeons declared that, whatever the condition, it was not attributable to any further mischief in the cæcum, as the abdomen was flaccid and without tenderness. At last it occurred to someone to look at the teeth, when several were found to be carious and in a most septic condition. It was dentistry that she needed; after the septic foci had been removed from the mouth the temperature fell to normal, and recovery was complete. Here again the influence of the oral condition upon the organism was not immediately appreciated. Is it not reasonable to believe that the original appendicitis had some connection with the septic teeth from infection from swallowing septic material? To me it appears more than probable that if there had been no carious teeth in the mouth there would have been no appendix abscess. I can adduce another case in support of this contention, this time in my own practice.

5. A few months ago I operated, in a nursing home, upon Miss X. for chronic appendicitis. There was no actual abscess, but the appendix was much inflamed, and buried amongst coils of intestine densely adherent. A large blood cyst of the right ovary, larger than a cricket ball, was removed through the lateral opening at the same time, but this has no bearing on the case. All went well until the fourth day, when the right parotid gland inflamed, with the subsequent formation of a deep-seated abscess, which I evacuated later. The temperature rose to 103° and 104°, and the patient was profoundly ill. The abdomen was quiescent throughout, the wound healing well by first intention. Now I come to the point I wish to emphasise. In this case I had neglected before the operation to examine the teeth. To all appearance, the patient had a beautiful set, the whiteness and evenness of those in front contributing in no small measure to her good looks; but I found out, to my chagrin, when the parotid abscess supervened, that there were no less than seven carious molars in this whited sepulchre of a mouth, harbouring and generating hordes of pathogenic organisms which, owing to the absence of solid food after the operation and the consequent stagnation and diminished secretion of saliva, multiplied exceedingly and doubtless infected directly the right parotid duct; for I think the theory of parotid abscess following laparotomy which now holds the field is that of direct infection of the gland from the mouth by way of Stenson's duct. Although the mouth teems with various micro-organisms of different degrees of potency and malevolence, yet the streptococcus pyogenes aureus is generally found to be predominant in cases of parotid abscess. Now, here again in this case it is curious to note how appendicitis supervened upon a septic condition of the mouth. Whether the micro-organisms of the appendix, the parotid, and the teeth were identical in this case I was unable to ascertain. I would recommend any one interested in Parotitis and Parotid abscess supervening upon abdominal section to refer to some back numbers of the *Annals of Surgery*. I cannot help thinking that the great prevalence of appendicitis in the present day bears a very definite relationship to the equally great prevalence of carious teeth. It is reasonable to believe that the appendix, being the weakest part of the intestinal tract—for it is often compared to a stagnant backwater—would be the first part to succumb to direct microbic infection from the intestinal contents; it is, moreover, a vestigial organ, and such are very prone to attack.

These few cases are only some out of very many—I could quote them indefinitely. They are taken as samples.

My experience in the Out-patient Department of this hospital is that dyspepsia, gastritis, and enteritis, when they are chronic, are closely connected with and are probably due to faulty dental conditions. If the teeth are absent mastication cannot be performed, and so the chemistry of the digestive ferments does not get a chance. If septic, then toxæmia, chronic or acute, may supervene; if the teeth are, some deficient and others septic, then both results described run concurrently, and this is the commonest state of affairs—a mixed and more complex condition. Since I have examined the teeth of my patients I have been surprised to find in how few cases good molars are approximated to good molars on both sides: if there are, for example, three good molars in the upper jaw on one side, there are none, perhaps, in the lower, and therefore they are useless. But is is better to have no teeth than septic teeth, and the edentulous are generally in a better physical condition than the possesors of septic mouths.

When I was a student we were required in case-taking to note carefully the condition of the heart and lungs, and any abnormalities of the abdominal viscera; we were directed to look at the tongue and inquire after the appetite; in some few cases we were advised to examine the fundus oculi with the ophthalmoscope, but never were we, to the best of my knowledge and belief, ever asked to examine the gums or teeth as a routine practice, and it was only in the dental department, or occasionally in the surgical out-patient department, that our attention was ever drawn to them. Now I believe much of this is altered. Many physicians insist that their clinical clerks should record in their case-books the condition of the teeth, and many surgeons refuse to operate until the teeth have been set in order.

The carious teeth should be stopped or extracted and the tartar should be removed before operation when practicable. A mouth-wash is a poor substitute for good dentistry, but one that we often perforce have to be content with. After operation the teeth and gums should be swabbed with cotton-wool on forceps by the nurse, the tongue should be scraped and the mouth constantly rinsed, and this attention to the mouth is, in my opinion, one of the most important duties of a nurse after severe operations, such as laparotomy. For the first few days, when solid food is not being taken, and the mouth is dry from the absence of salivary secretion, nothing conduces more to the comfort and well-being of the patient than constant attention to the mouth.

My own out-patient experience leads me to believe that if the mouths and teeth of the population were kept in a healthy state we should seldom hear of gastric and duodenal ulcer, and the entire large class of dyspepsias. There would be far less malnutrition and emaciation resulting from toxæmia and the ingestion of insufficiently masticated food. Appendicitis would be far less frequent. I hold the view that the increased prevalence of appendicitis as compared with 30 or 40 years ago, is due largely to the greater frequency of dental caries in our people. Whether dental caries is the cause or the result of the physical degeneration which exists so much at the present day is uncertain; it may be both cause and effect working in a vicious circle. It would be very instructive if in every case of appendicitis a careful note of the condition of the teeth were made. If this were done systematically we might be able to arrive at some definite conclusion as to the relationship between septic teeth and appendicitis.

Many years ago I lived among the Gauchos in the south of the Argentine Republic—a remarkably fine race physically, and the happy possessors of magnificent teeth. These people lived almost exclusively upon a meat diet, and after every meal it was their invariable habit to remove particles of food from between their teeth with their knives. This custom, though not an æsthetic one, doubtless tended, together with their diet, to preserve their teeth and their health, for, during the three years I was among them I never saw or heard of a case of appendicitis, and digestive troubles generally were practically unknown.

My experience of the Gauchos is paralleled by that of Dr. Perry, who was Government Medical Officer to the Siwash Indians of Vancouver Island, British Columbia, for eight years. I was greatly interested when he told me that all these people possessed excellent teeth; even the aged women, who could hardly walk from senile decrepitude, had no teeth missing. Dr. Perry states that several of these aged crones were reputed to be over 100 years old, so that it would appear that the possession of good teeth tends to longevity. He noticed amongst the Indians of Athabaska the same fact—the possession of excellent teeth. All these Indians live almost exclusively upon fish and game, just as the Gauchos of the Argentine Pampas live entirely on beef and mutton. My experience is that a flesh diet is conducive to the preservation of the teeth, and secondarily, to health.

I have occasionally, while riding to the north of Patagonia, come across skulls, probably of Indians, in which the teeth were all sound; and Dr. Perry tells me the same thing of the Indians of the North-West, and that in certain places, old battlefields, he has seen as many as forty or fifty skeletons lying together of Indian warriors who had fallen in battle; that he has often examined the skulls and found the teeth perfect. There are some skulls of the Siwash Indians in the Museum of the Royal College of Surgeons which exhibit this feature very well.

I am told by Colonel Lee, of the Indian Medical Service, who has spent the greater part of his life in Southern India, of a very different condition of affairs prevailing there. There the native diet is almost entirely farinaceous, and the habit is prevalent of chewing the areca nut, which is mixed with betel leaf, with a small addition of lime and tobacco. The teeth of these people are generally in a foul condition; no attempt is made to remove the tartar, which accumulates in great abundance, and pyorrhœa alveolaris is common. The Government hospitals and dispensaries are crowded with cases of chronic gastritis, chronic enteritis, a type of diarrhœa which is difficult to arrest, and febricula or continued fever of a mild character for which no cause can be assigned, but which not infrequently terminates fatally.

Contrast the two pictures. On the one hand the hardy, meat-eating Gauchos and Indians with their perfect teeth; on the other, the feeble Madrasi, with septic teeth and neglected mouths, living on cereals, a constant prey to dysentery and allied diseases. It would appear that "hard" food tends to preserve the teeth, while "soft" food disposes to dental caries and oral sepsis, which in turn may be followed by a whole train of secondary disturbances of the alimentary canal.

I trust I have said enough to emphasise the importance of this subject, which is not yet fully worked out.

